# A new species of *Dendropsophus* (Anura, Hylidae) from southwestern Amazonia with a green bilobate vocal sac

**DOI:** 10.3897/zookeys.942.51864

**Published:** 2020-06-18

**Authors:** Miquéias Ferrão, Jiří Moravec, James Hanken, Albertina Pimentel Lima

**Affiliations:** 1 Museum of Comparative Zoology, Harvard University, Cambridge, Massachusetts, USA Harvard University Cambridge United States of America; 2 Coordenação de Biodiversidade, Instituto Nacional de Pesquisas da Amazônia, Manaus, Amazonas, Brazil Instituto Nacional de Pesquisas da Amazônia Manaus Brazil; 3 Department of Zoology, National Museum, Prague, Czech Republic National Museum Prague Czech Republic

**Keywords:** Amphibia, advertisement call, Amazonian biodiversity, *Dendropsophus microcephalus* species group, *Dendropsophus bilobatus* sp. nov., integrative taxonomy, morphology, upper Madeira River

## Abstract

Recent studies have shown that species diversity of the South American frog genus *Dendropsophus* is significantly underestimated, especially in Amazonia. Herein, through integrative taxonomy a new species of *Dendropsophus* from the east bank of the upper Madeira River, Brazil is described. Based on molecular phylogenetic and morphological analyses, the new species is referred to the *D. microcephalus* species group, where it is differentiated from its congeners mainly by having a green bilobate vocal sac and an advertisement call comprising 1–4 monophasic notes emitted with a dominant frequency of 8,979–9,606 Hz. Based on intensive sampling conducted in the study area over the last ten years, the new species is restricted to the east bank of the upper Madeira River, although its geographic range is expected to include Bolivian forests close to the type locality.

## Introduction

The genus *Dendropsophus* Fitzinger, 1843 is a taxonomically difficult group of small and, for the most part, morphologically similar species. The group exhibits high species diversity – 108 species are currently recognized, of which 66 occur in Brazilian Amazonia (Segalla et al. 2016; Frost 2019) – as well as cryptic diversity due to a high degree of both phenotypic similarity among species and intraspecific polymorphism (Gehara et al. 2014; Caminer et al. 2017). Whereas traditional morphological methods have often failed to reveal cryptic species and accurately delimit species boundaries, non-morphological methods (e.g., molecular phylogenetics and bioacoustics) have proven to be very useful for reliably documenting the full extent of species diversity in the genus (e.g., Fouquet et al. 2015; Rivanadeira et al. 2018).

The advertisement call is the most common mate-recognition signal among anurans; it has a direct impact on sexual selection and speciation (e.g., Sullivan et al. 1995; Boul et al. 2006). Consequently, advertisement call characteristics are widely used to identify anuran species both in field-based faunal inventories and in taxonomic studies (Schneider and Sinsch 2007; Köhler et al. 2017). During herpetological surveys of the amphibian and reptile fauna in the vicinity of the upper Madeira River (southwestern Amazonia, Rondônia, Brazil) in 2011–2013, we recorded several anuran advertisement calls that were markedly different from calls of all described species of *Dendropsophus* known from Brazilian Amazonia. The frogs emitting these calls morphologically resemble members of the *D. microcephalus* species group (sensu Faivovich et al. 2005), and preliminary bioacoustic analyses revealed that their calls are monophasic, i.e., they consist of only one call type (e.g., Orrico et al. 2014), and have a remarkably high dominant frequency (above 8 kHz).

In the *Dendropsophus microcephalus* species group, a similarly high dominant frequency has been reported only for two “monophasic” species: *D. meridianus* (Lutz, 1954) and *D. ozzyi* Orrico, Peloso, Sturaro, Silva, Neckel-Oliveira, Gordo, Faivovich & Haddad, 2014 (Pombal and Bastos 1998; Orrico et al. 2014). A high dominant frequency (~ 9 kHz) was also reported for *D. minusculus* (Rivero, 1971) from Belem, Brazil, by Duellman and Pyles (1983), but a low dominant frequency (~ 3 kHz) was recorded by Tarano (2011) from a population of the same species closer to the type locality in Venezuela. We suspect that the report by Duellman and Pyles (1983) may represent a species misidentification, and that the population referred to *D. minusculus* instead likely corresponds to *D. ozzyi*.

We believe that the unknown *Dendropsophus* with high dominant frequency calls represent at least two new species, which differ markedly in body shape, coloration and molecular characters. Herein, we provide formal description of the most strikingly distinct of these species, which to date is known only from the east bank of the upper Madeira River. In addition to its distinctive advertisement call, the species is characterized by a green bilobate vocal sac. Our description combines morphological, bioacoustic and molecular data.

## Materials and methods

### Specimens examined

**Figure 1.**
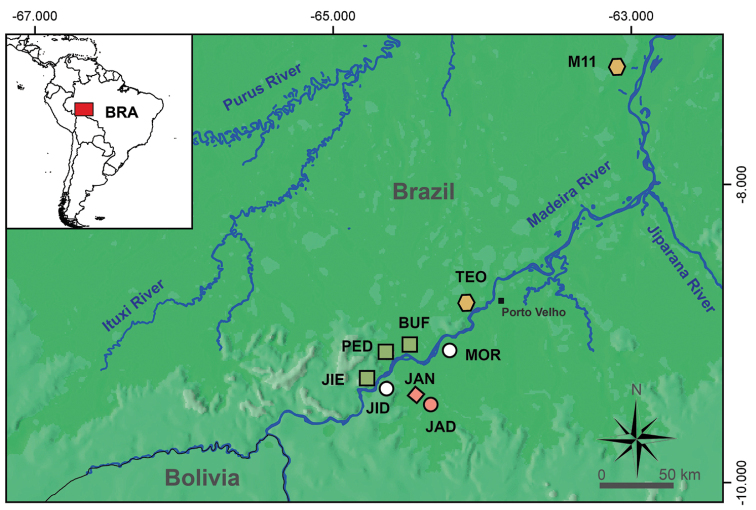
Schematic map showing RAPELD sampling sites in the upper Madeira River, Brazilian Amazonia. Symbols: green squares, *Dendropsophus* sp. A; orange pentagons, *Dendropsophus* sp. B; red diamond and circle, type and paratype localities of *Dendropsophus* sp. nov., respectively; white circles, referred specimens of *Dendropsophus* sp. nov. West bank: M11, Module 11; TEO, Teotônio; BUF, Bufalo; PED, Pedras; JIE, Jirau-Esquerdo. East bank: MOR, Morrinhos; JAD, Jaci-Direito; JAN, Jaci-Novo; JID, Jirau-Direito.

We examined adult specimens of three forms of *Dendropsophus* collected in nine long-term ecological research (hereafter RAPELD) sampling sites (Magnusson et al. 2013) on the east and west banks of the upper Madeira River (Fig. 1, Table 1). Collected individuals were killed by topical application of a 2% benzocaine solution. Tissue samples were then taken from all specimens and stored in 100% ethanol. Finally, all specimens were fixed in 10% neutral-buffered formalin and stored in 70% ethanol. Voucher specimens are deposited in the herpetological collection of the Instituto Nacional de Pesquisas da Amazônia, Manaus, Brazil (INPA-H). *Dendropsophus* species used for comparisons are listed in Appendix 1.

**Table 1.** RAPELD sampling sites in the upper Madeira River, Brazilian Amazonia.

Sampling site	Acronym	Geographic coordinates	Madeira River bank
Module 11	M11	07°13'06"S, 63°05'31"W	West
Teotônio	TEO	08°48'26"S, 64°05'56"W	West
Bufalo	BUF	09°09'32"S, 64°37'59"W	West
Pedras	PED	09°06'28"S, 64°30'46"W	West
Jirau-Esquerdo	JIE	09°17'52"S, 64°46'10"W	West
Jaci-Novo	JAN	09°24'45"S, 64°26'33"W	East
Jaci-Direito	JAD	09°27'44"S, 64°23'32"W	East
Jirau-Direito	JID	09°21'43"S, 64°41'31"W	East
Morrinhos	MOR	09°04'34"S, 64°14'46"W	East

### Morphological characters

The format for the description follows Moravec et al. (2008). Specimens were sexed based on the presence or absence of secondary sexual characters (e.g., vocal sac and vocal slits) in males. Morphometric measurements were taken to the nearest 0.1 mm using a dissecting microscope and digital calipers. Thirteen morphometric measurements follow Duellman (1970) and Heyer et al. (1990): SVL, snout–vent length; HL, head length; HW, head width; EN, eye–nostril distance; ED, horizontal eye diameter; TD, horizontal tympanum diameter; HAL, hand length; 3FD, third finger disk diameter; 4TD, fourth toe disk diameter; TL, tibia length; THL, thigh length; FL, foot length; TAL, tarsus length. Webbing formulae of toes follow Savage and Heyer (1967) as adapted by Myers and Duellman (1982). Field notes and photographs taken by A. P. Lima were used to describe coloration in life.

### Molecular analysis

We included samples of three forms of small-sized *Dendropsophus* collected in the area of the upper Madeira River during surveys in 2011–2013. The aim of those surveys was to detect phylogenetic diversity of *Dendropsophus* species distributed in this region. For the final dataset, we retrieved additional sequences of *Dendropsophus* from GenBank to locate phylogenetic positions of our new material in relation to DNA sequences published earlier, most importantly by Faivovich et al. (2005) and Jansen et al. (2011, 2019). We included species representing all *Dendropsophus* species groups (sensu Faivovich et al. 2005). Primary attention was paid to Amazonian species of the *D. microcephalus* species group. In concordance with earlier published phylogenies, we used *Xenohyla truncata* (Izecksohn, 1959) as an outgroup. The final dataset comprised 63 samples representing 34 nominal taxa, three new forms and the outgroup. All sequences acquired from GenBank are identified by GenBank accession numbers (Appendix 2).

Genomic DNA was extracted from muscle tissue of 16 specimens of the three new forms. DNA extractions were obtained using the Wizard Genomic DNA Purification Kit (Promega Corporation, USA) following the manufacturer’s protocols. We used the 16sbr (GCCGTCTGAACTCAGATCGCAT) and 16sar (CGCCTGTTTATCAAAAACAT) primers (Palumbi et al. 1991) to amplify a fragment of the 16S rRNA containing 495 base pairs (bp). The reaction conditions had a pre-heating step at 73 °C for 60 s, 35 cycles of denaturation at 92 °C for 10 s, primer annealing at 50 °C for 35 s, and primer extension at 72 °C for 90 s, followed by a final extension step of five minutes at 72 °C. PCR products were purified through Exonuclease I and Thermosensitive Alkaline Phosphatase (Thermo Fisher Scientific, USA) and followed ABI BigDye Terminator Cycle Sequencing Kit protocols (Life Technologies, USA) as recommended by the manufacturer. Amplicons were sequenced using the forward primer in Macrogen (Macrogen Inc., Seoul, Korea).

Sequences were visually checked and edited with GENEIOUS 7.1.7 (GeneMatters Corp, Minneapolis, MN, USA). The final 16S rRNA matrix was composed of 63 terminals and 495 bp. BIOEDIT (Hall 1999) was used to align the final matrix through the ClustalW algorithm (Thompson et al. 1994). The most probable evolutionary model explaining sequence divergence was estimated using the Akaike Information Criterion (AIC) in JMODELTEST 2.1.7 (Darriba et al. 2012), which recovered the GTR+G+I as the most probable evolutionary model.

Phylogenetic trees were inferred through Maximum Likelihood (ML). The ML phylogenetic tree was calculated under the GTRGAMMA model with IQTREE webserver (Trifinopoulos et al. 2016). Clade support was estimated through 5,000 ultrafast bootstrap approximation replicates. MEGA 6.06 (Tamura et al. 2013) was used in order to estimate the uncorrected-pairwise genetic distance (p-distance) and Kimura-2-Parameters genetic distance (K2P; Kimura 1980) between the new *Dendropsophus* forms and other members of the *D. microcephalus* species group included in the phylogenetic analyses.

### Bioacoustics

Advertisement calls of three males of the new *Dendropsophus* species (INPA-H 41302, 41303, 41304) were recorded in the sampling site Jaci-Novo during the rainy season on 15 February 2013. Calls were recorded with a Marantz PMD660 digital professional recorder (Marantz, Japan) and a Sennheiser K6/ME66 directional microphone (Sennheiser, Germany). The microphone was positioned approximately 1 m from each male. Recordings were made in wave format at a sampling rate of 44.1 kHz with 16-bits resolution. Air temperature taken with a digital thermometer during the recording was 25–26 °C (*N* = 3). Recordings are housed in the bioacoustic repository of the Amazonian Biodiversity Studies Centre at INPA (CENBAM 706, 707, 708).

Seven advertisement calls were analyzed for each recorded male. Advertisement call parameters were measured in RAVEN 1.5 (Bioacoustics Research Program 2015). Raven parameters were set as follows: window type = Blackman window, 3 dB filter bandwidth = 82 Hz, FFT window size = 2048 samples; FFT overlap = 80%, hop size = 4 ms. The following temporal and spectral parameters were inferred: call duration, inter-call interval, call period, number of notes, note duration, number of pulses per note, pulse duration, inter-pulse interval, dominant frequency (measured trough the function *Peak Frequency*), and bandwidth. The bandwidth was measured 20 dB below the peak frequency to avoid the overlap with background noise. Terminology of call measurements follows Köhler et al. (2017) while terminology of call structure follows Littlejohn and Harrison (1985). Graphic representation of the advertisement calls was produced in the R environment (R Core Team 2016) through the package seewave v.2.1 (Sueur et al. 2008). Seewave was set as follows: window = Hanning, FFT size = 150 samples, FFT overlap = 85%.

## Results

### Molecular analysis

**Figure 2.**
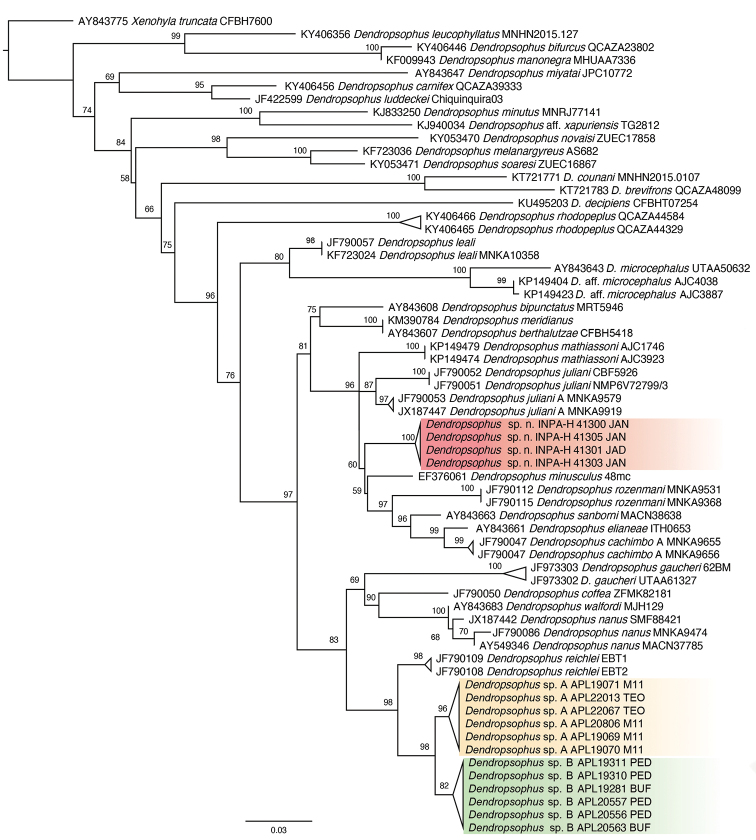
Maximum Likelihood phylogenetic tree of *Dendropsophus* inferred from the 16S rRNA mitochondrial gene (495 bp). Bootstrap values are shown close to nodes. Highlighted clades represent taxa from the upper Madeira River. Red horizontal bar denotes specimens from the east bank of the river; green and orange bars indicate specimens from the west bank.

The Maximum Likelihood (ML) analysis based on 16S rRNA recovers several well-supported clades within *Dendropsophus* (Fig. 2). Samples collected in the area of the upper Madeira River form three monophyletic lineages nested within a major clade (ML support = 96), which includes species of the *D. microcephalus* species group sensu Faivovich et al. (2005).

Specimens from the east bank of the river, which are characterized by high-pitched calls and a bilobate vocal sac of green color when deflated (hereafter referred as *Dendropsophus bilobatus* sp. nov.), are grouped in a well-supported clade (ML support = 96) consisting of *D. mathiassoni* (Cochran & Goin, 1970), *D. juliani* Moravec, Aparicio & Köhler, 2006, *D. juliani* A (sensu Jansen et al. 2011), *D. minusculus*, *D. rozenmani* Jansen, Santana, Teixeira & Köhler, 2019, *D. sanborni* (Schmidt, 1944), *D. elianeae* (Napoli & Caramaschi, 2000) and *D. cachimbo* A (sensu Jansen et al. 2011). Within this clade, *Dendropsophus bilobatus* sp. nov. is placed with low support (ML support = 60) as sister to the group formed by the last five of the above-mentioned species (Fig. 2). Interspecific pairwise genetic distances between *Dendropsophus bilobatus* sp. nov. and its close relatives range from 3.4 to 5.8% (p-distance) and 3.4 to 6.1% (K2P). The average intraspecific genetic distance is 0.3% (K2P and p-distance; Table 2).

**Table 2.** Interspecific pairwise genetic distances (expressed as a percentage and based on the 16S rRNA mitochondrial gene) between species of *Dendropsophus*, especially those in the *D. microcephalus* species group. Lower diagonal: uncorrected p-distance. Upper diagonal: Kimura-2-parameters (K2P).

	Species	1	2	3	4	5	6	7	8	9	10	11	12	13	14	15	16	17	18	19	20	21
1	*D. rhodopeplus*		8.0	11.7	10.2	10.5	11.3	10.2	9.2	9.2	9.3	9.2	9.9	9.1	9.4	9.3	7.4	10.7	9.1	9.1	8.4	9.0
2	*D. leali*	7.5		8.5	8.4	7.0	8.3	7.6	6.6	7.0	8.2	6.3	6.6	6.2	8.5	8.7	7.4	9.1	8.4	7.4	8.0	7.7
3	*D. gaucheri*	10.6	8.0		14.9	13.2	10	9.3	8.7	8.9	8.7	9.4	10.6	9.1	9.3	10.2	9.1	7.5	9.1	9.4	9.2	8.4
4	*D. microcephalus*	9.5	7.9	13.3		3.7	12.1	11	10.3	9.5	9.9	11.7	10.6	11.5	12.1	12	10.6	13	11.5	11.1	10.6	10.4
5	*D. microcephalus* COL	9.6	6.6	12	3.6		11.1	10.4	9.0	9.4	10.4	11	9.0	10.8	11.4	10.7	10.3	12.6	10.5	10.5	10.3	10.8
6	*D.* sp. A	10.4	7.8	9.2	11	10.2		1.7	3.1	6.8	6.8	7.6	7.5	7.6	8.8	8.7	9.1	10.5	9.1	8.2	8.2	7.8
7	*D.* sp. B	9.5	7.2	8.7	10.1	9.7	1.6		2.8	6.7	6.2	7.0	6.7	6.9	7.7	7.5	8.7	10.4	8.2	7.5	7.7	7.5
8	*D. reichlei*	8.6	6.3	8.1	9.6	8.4	3.1	2.8		5.0	5.8	5.8	6.5	5.7	7.0	6.7	7.1	8.3	7.3	6.3	6.6	7.0
9	*D. coffea*	8.6	6.6	8.3	8.9	8.8	6.4	6.3	4.8		4.7	4.9	7.2	4.8	7.8	8.1	7.0	9.2	8.6	7.1	6.5	7.1
10	*D. nanus/walfordi*	8.7	7.7	8.1	9.2	9.7	6.4	5.9	5.5	4.5		6.0	7.1	5.8	7.3	7.9	6.7	7.4	7.5	6.2	5.5	6.5
11	*D. meridianus*	8.6	6.0	8.7	10.7	10.1	7.2	6.6	5.6	4.7	5.7		4.6	2.0	6.8	5.4	5.2	6.0	6.6	5.3	4.9	6.1
12	*D. bipunctatus*	9.2	6.3	9.8	9.8	8.4	7.1	6.4	6.2	6.8	6.7	4.4		4.8	6.4	7.6	5.8	7.2	6.2	5.8	4.9	6.0
13	*D. berthalutzae*	8.5	5.9	8.5	10.6	9.9	7.2	6.6	5.4	4.6	5.6	2.0	4.6		6.6	5.3	5.0	5.7	6.4	5.2	4.7	6.0
14	*D. cachimbo* A	8.8	8.0	8.7	11.1	10.5	8.2	7.3	6.6	7.4	6.9	6.4	6.1	6.3		5.4	5.2	6.0	3.4	4.8	4.7	5.2
15	*D. rozenmani*	8.7	8.2	9.5	11	9.9	8.2	7.1	6.3	7.6	7.4	5.2	7.1	5.1	5.1		5.9	5.8	4.7	4.8	5.1	6.1
16	*D. mathiassoni*	7.0	7.0	8.5	9.8	9.5	8.5	8.1	6.7	6.6	6.4	4.9	5.5	4.8	5.0	5.6		4.0	4.8	4.6	3.3	4.7
17	*D. minusculus*	9.9	8.5	7.1	11.8	11.4	9.7	9.6	7.8	8.5	7.0	5.7	6.8	5.4	5.7	5.6	3.9		4.9	4.9	4.1	4.8
18	*D. sanborni*	8.5	7.9	8.5	10.6	9.7	8.5	7.7	6.9	8.1	7.0	6.2	5.9	6.1	3.3	4.5	4.6	4.7		4.6	3.2	4.1
19	*D. juliani*	8.5	7.0	8.7	10.2	9.7	7.7	7.1	6.0	6.7	5.9	5.1	5.5	5.0	4.6	4.7	4.4	4.7	4.4		2.4	4.2
20	*D. juliani* A	7.9	7.5	8.6	9.8	9.6	7.7	7.2	6.3	6.2	5.3	4.7	4.7	4.5	4.5	4.9	3.2	3.9	3.2	2.3		3.4
21	*D. bilobatus* sp. nov.	8.4	7.3	7.8	9.6	9.9	7.4	7.1	6.7	6.8	6.2	5.8	5.7	5.7	5.0	5.8	4.5	4.6	3.9	4.1	3.4	

*Dendropsophus* specimens from the west bank of the upper Madeira River cluster in sister position to *D. reichlei* Moravec, Aparicio, Guerrero-Reinhard, Calderon & Köhler, 2008 from Bolivia (ML support = 98). These frogs sort into two well-supported sister lineages (ML support = 98; Fig. 2). The first lineage (hereafter referred to as *Dendropsophus* sp. A) comprises specimens collected in the RAPELD Teotonio and M11 sampling sites (distance apart ~ 250 km). The second lineage (*Dendropsophus* sp. B) comprises specimens from the RAPELD Pedras and Bufalos sampling sites (distance apart ~ 20 km). Genetic distances between *D.* sp. A and *D.* sp. B range from 1.6% (p-distance) to 1.7% (K2P). Genetic distances between *D. reichlei* and *D.* sp. A (K2P and p-distance = 3.1%) are slightly higher than those between *D. reichlei* and *D.* sp. B (K2P and p-distance = 2.8%). The average intraspecific genetic distance is higher in *Dendropsophus* sp. A (K2P and p-distance = 0.4%) than in *D.* sp. B (K2P and p-distance = 0.1%).

Because *Dendropsophus bilobatus* sp. nov. also differs from other congeneric species by its remarkably distinct morphology, we here describe it as a new species. Resolution of the taxonomic status of *D.* sp. A and *D.* sp. B is pending the results of additional species delimitation tests, which will be treated in a future study.

### Taxonomy

#### Dendropsophus bilobatus sp. nov.

Taxon classificationAnimaliaAnuraHylidae

4C8E0309-3D45-5CED-A407-38C10A2B8CB4

http://zoobank.org/18906B0C-5EEA-416B-A672-FF8AD98DA448

**Figure 3.**
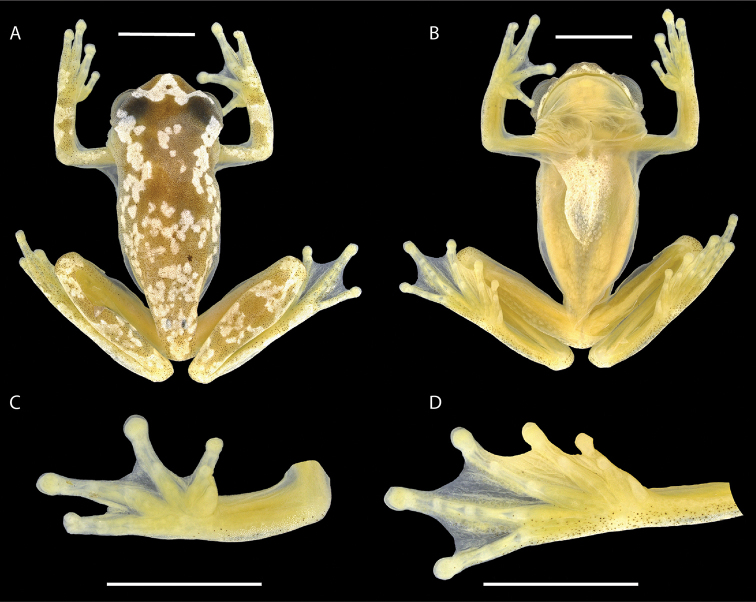
Dorsal view of the body (**A**) and ventral views of the body (**B**), hand (**C**) and foot (**D**) of the preserved holotype of *Dendropsophus bilobatus* sp. nov. (INPA-H 41300) from the RAPELD Jaci-Novo sampling site, east bank of the upper Madeira River, municipality of Porto Velho, Rondônia, Brazil. Scale bars: 5 mm. Photographs: Jeni Lima Magnusson.

**Figure 4.**
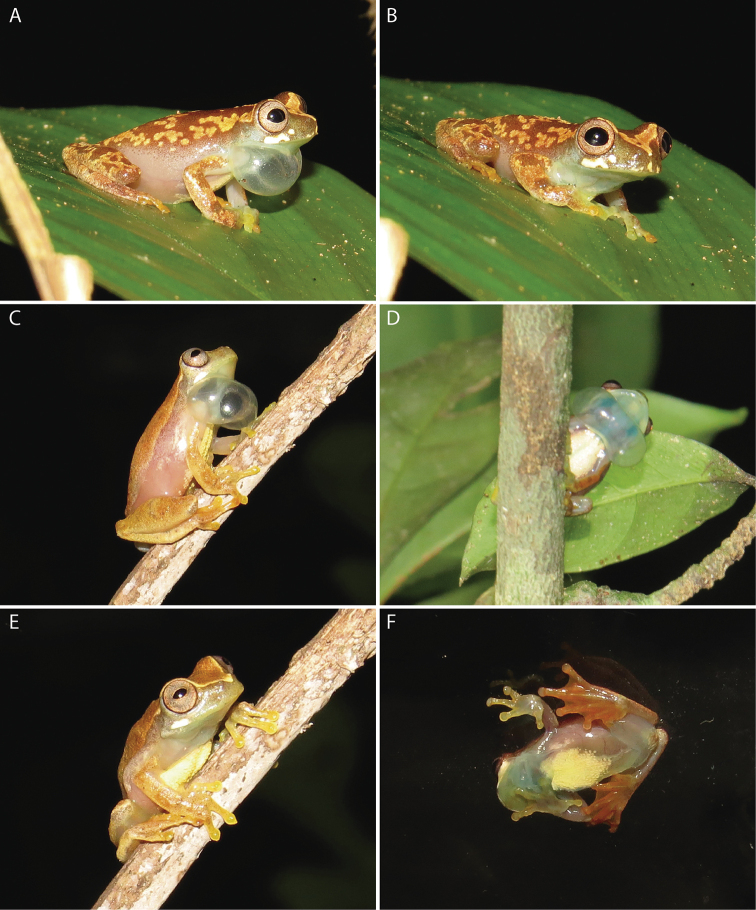
*Dendropsophus bilobatus* sp. nov. from the Jaci-Parana River, a tributary of the east bank of the upper Madeira River, municipality of Porto Velho, Rondônia, Brazil, in life. **A, B** Holotype, INPA-H 41300, SVL = 18.8 mm, Jaci-Novo sampling site. **C–E** Adult male, INPA-H 41303, SVL = 19.9 mm, Jaci-Novo sampling site. **F** Adult male, INPA-H 41301, SVL = 18.9 mm, Jaci-Direito sampling site. Note the inflated translucent greenish bilobate vocal sac (**A, C, D**). Photographs: Albertina Pimentel Lima.

**Figure 5.**
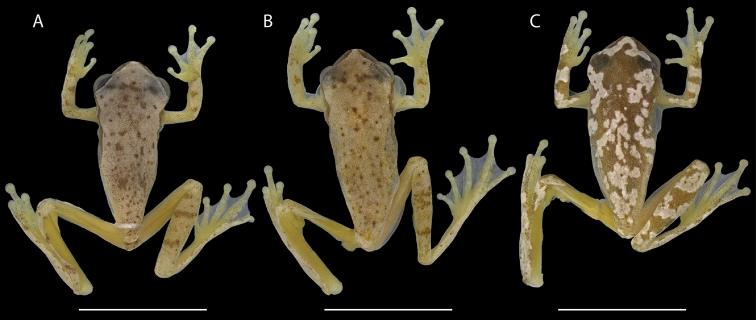
Preserved males of *Dendropsophus bilobatus* sp. nov. in dorsal view showing color variation. **A**INPA-H 41302, SVL = 19.4 mm **B**INPA-H 41304, SVL = 20.1 mm **C**INPA-H 41306, SVL = 20.8 mm. Scale bars: 10 mm. Photographs: Jeni Lima Magnusson.

**Figure 6.**
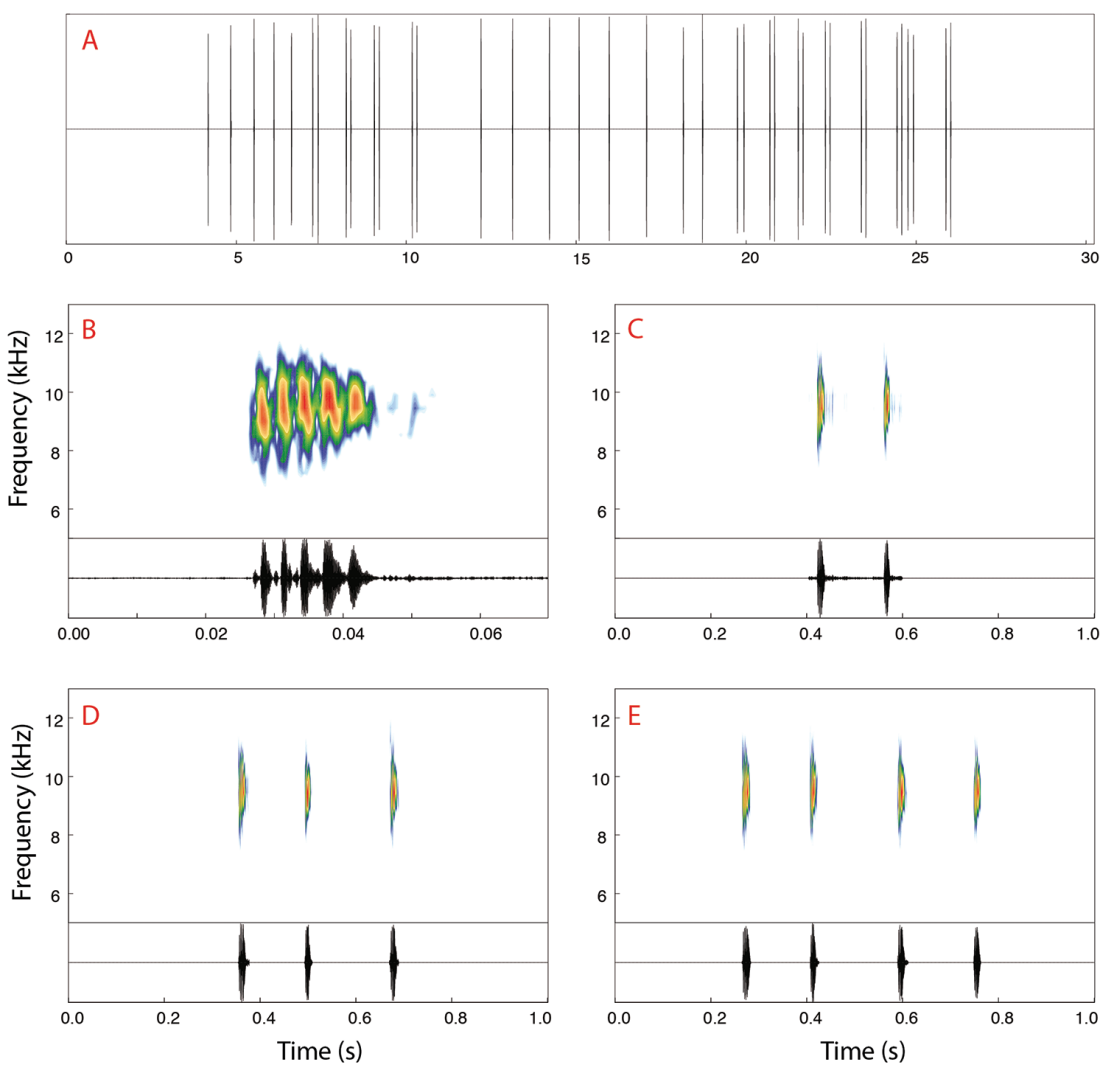
Advertisement call of *Dendropsophus bilobatus* sp. nov. from the RAPELD Jaci-Novo sampling site, Jaci-Parana River, a tributary of the east bank of the upper Madeira River, municipality of Porto Velho, Rondônia, Brazil. **A** Oscillogram of a call series composed of 24 calls. Spectrograms (upper graphs) and oscillograms (lower graphs) of calls formed by **B** one **C** two **D** three and **E** four pulsed notes. Recorded male: INPA-H 41303. Air temperature: 25.7 °C.

Figures 2 , 3 , 4 , 5 , 6 , Table 2 , 3

##### Material.

***Holotype.***INPA-H 41300 (field number APL 19703; GenBank accession number MN977837), an adult male from the RAPELD Jaci-Novo sampling site (09°24'45"S, 64°26'33"W; 117 m a.s.l.), flooded forest at the west bank of the Jaci-Parana River (east tributary of the upper Madeira River), municipality of Porto Velho, district of Jaci-Parana, state of Rondônia, Brazil, collected on 26 March 2013 by Albertina P. Lima.

***Paratopotypes.*** Five males: INPA-H 41302 (field number APL 19442), 41303 (field number APL 19443; GenBank accession number MN977835), 41304 (field number APL 19444), 41305 (field number APL 19445; GenBank accession number MN977836), and 41306 (field number APL 19446), collected on 15 February 2013 by Albertina P. Lima.

***Paratypes.*** Two males: INPA-H 41301 (field number APL 19419; GenBank accession number MN977834) and 41307 (field number APL 19448), from the RAPELD Jaci-Direito sampling site (09°27'44"S, 64°23'32"W; 121 m a.s.l.), east bank of the Jaci-Parana River (an east tributary of the upper Madeira River), municipality of Porto Velho, district of Jaci-Parana, state of Rondônia, Brazil, collected on 14 and 15 February 2013, respectively, by Albertina P. Lima.

***Referred material.*** Three males: INPA-H 41308 (field number APL 16652) and 41309 (field number APL 16653), from the RAPELD Jirau-Direito sampling site (09°21'43"S, 64°41'31"W; 131 m a.s.l.), east bank of the upper Madeira River, municipality of Porto Velho, state of Rondônia, Brazil, collected on 20 January 2011 by Albertina P. Lima; and INPA-H 41310 (field number APL 16428), from the RAPELD Morrinhos sampling site (09°04'34"S, 64°14'46"W; 95 m a.s.l.), municipality of Porto Velho, state of Rondônia, Brazil, collected on 13 January 2011 by Albertina P. Lima.

##### Generic placement.

We assign this species to *Dendropsophus* based on our molecular phylogenetic analysis (Fig. 2) and on its general morphological similarity to other members of the genus.

##### Diagnosis.

A species of the *Dendropsophus microcephalus* species group, distinguished from other species of *Dendropsophus* by the following combination of characters: (1) small size, SVL 18.8–20.8 mm (*N* = 8) in males (females unknown), head slightly wider than body; (2) snout short, truncate in dorsal and lateral views; (3) tympanum evident, round, about one third of eye diameter, tympanic annulus distinct anteriorly, ventrally and partly posteriorly; supratympanic fold barely evident; (4) dentigerous processes of vomers small, barely prominent, and separated medially between posterior halves of choanae; (5) skin on dorsal surfaces smooth; (6) tarsal fold and tubercles on outer edge of tarsus absent; ulnar folds and tubercles absent; (7) axillary membrane extensively developed; (8) fingers about half webbed; toes about three-fourths webbed; (9) bifid distal subarticular tubercle under fourth finger; (10) pectoral glands absent; (11) generally darker coloration of the loreal-tympanic region contrasts sharply with the lighter dorsal head coloration, one or two white spots below the eye; (12) in life, ground coloration of dorsum light brown; head greenish brown laterally; flanks ventrally and posteriorly a translucent pinkish white without chromatophores; hidden surfaces of thighs yellow without melanophores; (13) in life, throat green in males; belly yellowish-white in pectoral and central parts, translucent pinkish-white in posterior and lateral parts; ventral surfaces of thighs translucent pinkish white; (14) in life, iris pale to dark brown with barely visible tiny brown veins, iris periphery dark brown to black; bones white; (15) advertisement call consisting of 1–4 notes (usually 1–2 notes), emitted regularly in series of 7–35 calls; high-pitched, monophasic, pulsed notes (3–8 pulses) with a duration of 12–24 ms and a dominant frequency of 8,979–9,606 Hz.

##### Comparisons.

*Dendropsophus bilobatus* sp. nov. is readily distinguished from all congeners by having a green bilobate subgular vocal sac (some members of the *D. marmoratus* species group have a bilobate vocal sac, but not green) and a monophasic advertisement call with a remarkably high dominant frequency (8,979–9,606 Hz). Below we describe additional important differences between the new species and other members of the *D. microcephalus* species group (sensu Faivovich et al. 2005) that occur in Brazilian Amazonia and surrounding areas of Bolivia, Colombia, Peru and Ecuador. Characters of *D. bilobatus* are set in parentheses if not otherwise stated.

Three species of the *Dendropsophus microcephalus* species group have advertisement calls with a high dominant frequency: *D. meridianus* (Lutz, 1954), *D. minusculus*, and *D. ozzyi*. However, *D. meridianus* differs from *D. bilobatus* in having a snout slightly acuminate in lateral view (truncate), a single subgular yellow vocal sac (bilobate, green), dark dorsal lines or stripes on the dorsum (absent), absence of white subocular spots (present; Lutz 1973), and the dominant frequency of the advertisement call reaches 8,000 Hz (it reaches 9,606 Hz in *D. bilobatus* ; Lutz 1973, Pombal and Bastos 1998); *D. minusculus* can be distinguished by its yellow single subgular vocal sac (bilobate, green) and by the absence of white subocular spots (present; Zina et al. 2014); and *D. ozzyi* differs in its single subgular transparent vocal sac (bilobate, green), absence of white subocular spots (present), vivid orange palmar end plantar surfaces (palmar surface greenish yellow, plantar surface orange), webbing formula of feet I 2–2+ II 1^+^–3- III 1^+^–2^+^ IV 2^+^–1^+^ V (I 1^+^–2- II 1^+^–1^1/2^ III 1^1/2^–2- IV 2-–1^+^ V), presence of glandular structures restricted on toes III and IV (glandular structures present also on toes II and V), and in single notes of the advertisement call (pulsed notes; Orrico et al. 2014).

The dark-greenish-brown coloration of the loreal-tympanic region of *Dendropsophus bilobatus* , which sharply contrasts with the light brown dorsal head coloration, resembles the head color pattern of *D. coffea* (Köhler, Jungfer & Reichle, 2005), *D. cruzi* (Pombal & Bastos, 1998), *D. studerae* (Carvalho-e-Silva, Carvalho-e-Silva & Izecksohn, 2003), *D. juliani*, *D. meridianus*, *D. microcephalus* (Cope, 1886), *D. minusculus*, *D. shiwiarum* Ortega-Andrade & Ron, 2013, *D. tintinnabulum* (Melin, 1941), and *D. reichlei*, but the new species is easily distinguished from each named species as follows (species already distinguished above are not listed here): *D. coffea* lacks white subocular spots (present) and has dark brown dorsal stripes (absent; Köhler et al. 2005); in *D. cruzi*, the thigh is longer than the tibia (tibia longer than thigh; Pombal and Bastos 1998); *D. studerae* has tuberculate dorsal skin (smooth; Carvalho-e-Silva et al. 2003); *D. juliani* has an acutely rounded snout in dorsal view (truncate), absence of white subocular spots (present), and greenish yellow plantar surfaces (orange; Moravec et al. 2006); *D. microcephalus* has maximum male SVL 24.5 mm (20.8 mm), an acutely rounded snout in dorsal view (truncate), an ovoid tongue (cordiform), and a weak tarsal fold (absent; Duellman 1970); *D. shiwiarum* has the discs of finger III and toe IV with pointed tips (pointed tips absent), a prominent conical tubercle on the dorsal surface of fingers III and IV (tubercle absent), both palmar and plantar surfaces unpigmented (palmar surface greenish yellow, plantar surface orange), and a lower dominant frequency of the advertisement call (3,984–5,254 Hz in *D. shiwiarum* vs. 8,979–9,606 Hz in *D. bilobatus* ; Ortega-Andrade and Ron 2013); *D. tintinnabulum* has a triangular-to-rounded snout in dorsal view (truncate) and orange palmar surfaces (greenish; Teixeira and Giaretta 2017), and lacks white subocular spots (present); and *D. reichlei* has a rounded snout in dorsal view (truncate) and a distinct canthus rostralis (absent), and lacks a glandular nuptial pad (present; Moravec et al. 2008).

In our phylogenetic analysis, the clade that contains *Dendropsophus bilobatus* is closely related to *D. bipunctatus* (Spix, 1824), *D. meridianus* and *D. berthalutzae* (Bokermann, 1962) from the southern and southeastern Brazilian coast (Fig. 2). In addition to differences in shape and color of the vocal sac and in advertisement call, these three species can be distinguished from *D. bilobatus* as follows: *D. bipunctatus* has a granulate dorsum (smooth), maximum SVL in males 25 mm (maximum male SVL 20.8 mm) and several small spots surrounded by a dark network that are distributed across the subocular area and lateral snout (spots only on subocular area and are not surrounded by dark network; Lutz 1973); *D. berthalutzae* has a snout that is slightly mucronate in dorsal view (truncate) and longer than eye diameter (snout shorter than eye diameter), and a minute outer metatarsal tubercle (absent; Lutz 1973).

Nine other small Amazonian species have been associated with the *Dendropsophus microcephalus* species group. These species differ from *D. bilobatus* in having the following combinations of characters: *D. joannae* (Köhler & Lötters, 2001) has tuberculate dorsal skin (smooth), a red inner iris in life (iris light to dark brown), and uniform head coloration without white subocular spots (coloration of loreal-tympanic region sharply outlined, subocular spots present; Köhler and Lötters 2001); *D. leali* (Bokermann, 1964) has a uniform ground head coloration without white subocular spots (coloration of loreal-tympanic region sharply outlined, subocular spots present: Köhler and Lötters 2001) and a biphasic call (monophasic; A. P. Lima personal data); *D. haraldschultzi* (Bokermann, 1962), *D. nanus* (Boulenger, 1889), *D. sanborni* (Schmidt, 1944) and *D. walfordi* (Bokermann, 1962) have a more pointed snout (snout short, truncate in dorsal and lateral views), a more or less conspicuous pattern of numerous thin brown lines on a yellowish dorsum (lines absent) and a biphasic call (monophasic; Hödl 1977; Teixeira et al. 2016; Missassi et al. 2017); *D. mathiassoni* (Cochran & Goin, 1970) has dorsolateral lymphatic sacs (absent; Cochran and Goin 1970); *D. rhodopeplus* (Günther, 1858) has a yellow dorsum with bright purple or red marks (purple or red marks absent; Duellman 2005); and *D. riveroi* (Cochran & Goin, 1970) has a canthus rostralis (absent) but lacks glandular nuptial pads in males (present; Ortega-Andrade and Ron 2013).

Ten other small Amazonian species belong to the *Dendropsophus rubicundulus* clade of the *D. microcephalus* species group (sensu Faivovich 2005). These species can be distinguished from *D. bilobatus* as follows: *D. anataliasiasi* (Bokermann, 1972), *D. araguaya* (Napoli & Caramaschi, 1998), *D. cerradensis* (Napoli & Caramaschi, 1998), *D. jimi* (Napoli & Caramaschi, 1999), *D. rhea* (Napoli & Caramaschi, 1999), *D. rozenmani*, *D. rubicundulus* (Reinhardt & Lütken, 1862) and *D. tritaeniatus* (Bokermann, 1965) lack white subocular spots (present) and have conspicuous dark brown stripes or small dark brown spots arranged in longitudinal lines on the dorsum (dorsum with irregular pattern of irregular yellow spots or small dark brown dots; Martins and Jim 2004, Teixeira et al. 2013, Teixeira and Giaretta 2015, Jansen et al. 2019); and *D. cachimbo* (Napoli & Caramaschi, 1999) and *D. elianeae* have a uniformly green or yellowish green dorsum (dorsum light brown with irregular pattern of yellow spots or small dark brown dots) and lack white subocular spots (spots present; Jansen et al. 2019).

The two unnamed forms of *Dendropsophus* in the *D. microcephalus* species group from the west bank of the upper Madeira River (*D.* sp. A and *D.* sp. B) differ from *D. bilobatus* in having a single yellow subgular vocal sac (bilobate, green) and pointed discs on toes and fingers (rounded).

Five *Dendropsophus* species distantly related to the *D. microcephalus* species group are reported from the area of the upper Madeira River (A. P. Lima personal data): *D. kamagarini* Rivadeneira, Venegas & Ron, 2018, *D. koechlini* (Duellman & Trueb, 1989), *D. leucophyllatus* (Beireis, 1783), *D. minutus* (Peters, 1872) and *D. sarayacuensis* (Shreve, 1935). These species differ clearly in their larger size and coloration (Rodriguez and Duellman 1994, Peloso et al. 2016, Caminer et al. 2017, Rivadeneira et al. 2018).

Currently, *Dendropsophus amicorum* (Mijares-Urrutia, 1998), *D. battersbyi* (Rivero, 1961), *D. bromeliaceus* Ferreira, Faivovich, Beard, & Pombal, 2015 and *D. yaracuyanus* (Mijares-Urrutia & Rivero, 2000) are not assigned with certainty to any species group. However, *Dendropsophus bilobatus* differs from *D. amicorum*, *D. battersbyi* and *D. yaracuyanus* by the SVL in males of 18.8–20.8 mm in males (SVL 22.8 mm in the male holotype of *D. amicorum*, SVL 33 mm in the male holotype of *D. battersbyi*, SVL 28.5–30.4 mm in males of *D. yaracuyanus*; Rivero 1961, Mijares-Urrutia 1998, Mijares-Urrutia and Rivero 2000); from *D. bromeliaceus* by the presence of subocular spots and webbing formula of fingers I 2^+^–2 II 1^1/2^–2^2/3^ III 2- –2 IV (subocular spots absent, I trace II 2-–3- III 3^+^–3^+^ IV; Ferreira et al. 2015). Although *Dendropsophus minimus* (Ahl, 1933) was placed in the *D. minimus* species group (sensu Faivovich et al. 2005), this species has never been included in a phylogenetic analysis and its group membership is uncertain. *Dendropsophus bilobatus* can be distinguished from *D. minimus* by having a visible tympanum and by the absence of tarsal fold (concealed tympanum and presence of tarsal fold; Ahl 1933).

##### Holotype description.

INPA-H 41300. Adult male (Figs 3, 4A, B), SVL 18.8 mm; body moderately robust; head slightly wider than long (HW/HL = 1.08); snout truncate in dorsal and lateral views; snout short, eye-nostril distance shorter than eye diameter (END/ED = 0.68); canthus rostralis rounded in dorsal and lateral views; loreal region slightly concave; internarial area slightly depressed; nostrils barely protuberant, directed dorsolaterally; interorbital area flat, slightly depressed in the central portion; interorbital distance equal 34% of head width; eyes large, strongly protuberant, ED/TD = 3.30, ED/HL = 0.42; tympanic membrane small, round, clearly distinct, its diameter 30% of eye diameter and 13% of head length; tympanic annulus distinct ventrally and anteriorly; supratympanic fold barely evident, slightly obscuring the upper edge of the tympanum. Arms slender and not hypertrophied; ulnar tubercles and fold absent; axillary membrane reaches the second third of the upper arm; hand relatively long, about 30% of SVL, approximately the same size as the forearm; fingers long, slender, bearing small discs; finger III twice as wide medialy than anteriorly; relative length of fingers I<II<IV<III; discs rounded on fingers; diameter of disc on finger III about the size of the tympanum; subarticular tubercles of fingers I and IV medium to large-sized, round, prominent, bifid in finger IV; subarticular tubercles of fingers II–III small, round, prominent; supernumerary tubercles barely evident; palmar tubercle small, flat, oval, barely evident proximally; prepollical tubercle large, flat, ovoid; nuptial pad white, glandular, covering the dorsolateral portion of the thumb but not reaching the ventral surface; webbing formula of fingers I 2^+^–2 II 1^1/2^–2^2/3^ III 2- –2 IV. Legs moderately long, slender (THL/SVL = 0.55; TL/SVL = 0.56); tibia slightly longer than thigh (TL/THL = 1.02); tarsal fold and tarsal tubercles absent; calcar tubercles absent; toes moderately long, bearing discs slightly smaller than those on fingers; toe IV length equals 60% of foot length; relative length of toes I<II<III<V<IV; toes I, II and V slender; toes III and IV widened by elongated flat glandular structures on both sides, glandular structures forming a continuous elongated glandular patch along toe IV, small glandular aggregations present also on fingers II and V; discs rounded on toes; diameter of the disc on toe IV equals diameter of the disc on finger III; subarticular tubercles round, prominent, penultimate tubercle on toe V bifid; supernumerary tubercles on toes III–IV small, round, barely evident; inner metatarsal tubercle elliptical, flat; outer metatarsal tubercle barely distinct; webbing formula of toes I 1^+^–2- II 1^+^–1^1/2^ III 1^1/2^–2- IV 2-–1^+^ V.

Skin on head, dorsum, dorsal surfaces of limbs and flanks smooth; vocal sac and ventral surfaces of arms smooth; belly smooth laterally, coarsely granular medially; lower surfaces of thighs and surroundings of cloaca slightly granular. Cloacal opening directed posteroventrally at midlevel of thigh, covered dorsally by a wide cloacal sheath. Choanae small, vertically oval; dentigerous processes of vomers small, three vomerine teeth present on the right process, absent on the left process. Tongue cordiform, posterior third not attached to the floor of the mouth. Vocal slits long, extending from the midlateral base of the tongue to the angle of the jaw; anterior part covered by the lateral margin of the tongue. Vocal sac bilobate, subgular (Figs 3A, 4A, C, D).

In life (Fig. 4A, B), the dorsum and dorsal surfaces of the limbs are light brown with an irregular pattern of yellow spots; the head has a large triangular yellow blotch that extends from the tip of the snout to the anterior interorbital region, including the anterior margin of the upper eyelids; the lateral sides of the head are greenish brown with two white horizontally elongate subocular spots on the left side and one elongate and one round white spot on the right side. The iris is pale to dark brown with barely visible tiny brown veins; its outer edge is brown to black. Proximal dorsal surfaces of fingers I–III are greenish white to yellowish white; the proximal dorsal surface of finger IV is brown; distal dorsal surfaces of the fingers are yellowish orange; nuptial pads are white. The upper part of the flanks is a light pinkish brown; the posterior part of the flanks and the groin are pinkish white. Hidden dorsal surfaces of the thighs are yellow. The vocal sac is green when deflated but translucent greenish white when inflated. The chest and belly are yellowish white medially but translucent pinkish white laterally and posteriorly. Ventral surfaces of arms and legs are translucent pinkish white; the anteroventral side of the thigh is yellow, the posteroventral side is pinkish white; palmar surfaces are greenish yellow; plantar surfaces are orange. Bones are white.

In alcohol (Fig. 3), the head and dorsum are cream to brown with numerous tiny black melanophores and irregular white spots and blotches; dorsal surfaces of the limbs are light cream or translucent; ventral surfaces are translucent to cream, the chest and medial area of the belly are white. Bones are white.

Holotype measurements (in mm): SVL, 18.8; HL, 6.1; HW, 6.6; EN, 1.7; ED, 2.5; IOD, 2.3; TD, 0.8; 3FD, 0.8; 4TD, 0.8; TL, 14.4; THL, 10.3; TAL, 5.6; FL, 14.1.

##### Variation.

The morphology of paratypes and paratopotypes does not deviate from that of the holotype. Morphometric measurements of all type specimens are shown in Table 3. *Dendropsophus bilobatus* sp. nov. exhibits two dorsal color patterns. The pattern of the holotype, while less common, is shared with two other specimens (INPA-H 41306 and 41307). The second and most common pattern is characterized by the dorsum and dorsal surfaces of the limbs being light brown with small irregularly distributed brown dots (Fig. 5A, B). Different from the holotype’s pattern (Fig. 5C), the limit between the pinkish flanks and the light brown dorsum is well marked (Fig. 4C). The number of subocular light spots is variable in both patterns, ranging from 1 to 3 spots. White nuptial pads are conspicuous in all specimens but absent in paratopotype INPA-H 41306. Ventral color is similar in all specimens, as well as the color of the bilobate vocal sac. Females are unknown.

**Table 3.** Morphometric measurements of the type series of *Dendropsophus bilobatus* sp. nov. from the east bank of the Madeira River, Municipality of Porto Velho, Rondonia, Brazil. Bold font denotes the holotype. Abbreviations: Desv., standard deviation, Min., minimum, Max., maximum. Morphometric abbreviations are described in Materials and methods.

Voucher	SVL	HL	HW	EM	ED	IOD	TD	3FD	4TD	TL	THL	TAL	FL
**INPA-H 41300**	**18.8**	**6.1**	**6.6**	**1.7**	**2.5**	**2.3**	**0.8**	**0.8**	**0.8**	**10.4**	**10.3**	**5.6**	**8.4**
INPA-H 41304	20.1	6.9	7.2	1.8	2.8	2.5	0.9	1.0	0.9	11.1	10.7	6.1	9.2
INPA-H 41305	19.4	6.4	6.8	1.9	2.6	2.4	0.9	0.9	0.9	10.7	10.2	6.1	9.0
INPA-H 41303	19.9	5.8	6.6	1.6	2.4	2.4	1.9	0.9	1.0	10.2	10.2	5.9	8.8
INPA-H 41301	18.9	6.3	6.4	1.6	2.5	2.2	0.9	0.8	0.8	10.2	9.7	5.6	8.1
INPA-H 41307	19.6	6.7	7.0	1.8	2.7	2.4	0.8	1.0	1.0	11.2	11.0	6.2	9.5
INPA-H 41306	20.8	6.6	6.8	1.7	2.8	2.1	1.0	0.9	0.9	11.5	10.9	6.0	9.4
INPA-H 41302	19.4	6.5	6.7	1.9	2.9	2.2	0.9	0.8	1.0	10.5	9.5	5.5	8.6
Mean	19.6	6.4	6.8	1.7	2.6	2.3	1.0	0.9	0.9	10.7	10.3	5.9	8.9
Desv.	0.6	0.3	0.2	0.1	0.2	0.1	0.4	0.1	0.1	0.5	0.5	0.3	0.5
Min.	18.8	5.8	6.4	1.6	2.4	2.1	0.8	0.8	0.8	10.2	9.5	5.5	8.1
Max.	20.8	6.9	7.2	1.9	2.9	2.5	1.9	1.0	1.0	11.5	11.0	6.2	9.5

##### Call description.

The advertisement call of *Dendropsophus bilobatus* (Fig. 6) consists of single- or multiple-note calls emitted regularly in series of 7–35 calls (19 ± 9, *N* = 12). The most common arrangements are the single-note call (*N* = 181) and the two-note call (*N* = 58), while the rarest are the three-note (*N* = 1) and four-note calls (*N* = 1). Single-note calls have a call duration of 12–24 ms (8.2 ± 3, *N* = 30), an inter-call interval of 483–1,284 ms (751 ± 201, *N* = 30), and a call period of 503–1,302 ms (769 ± 202, *N* = 30). Two-note calls have a call-duration of 155–199 ms (171 ± 13, *N* = 22), an inter-call interval of 437–1,347 ms (816 ± 196, *N* = 19), and a call period of 612–1,542 ms (985 ± 198, n = 19). Notes in the two-note calls have a note duration of 12–22 ms (17 ± 3, *N* = 44) and an inter-note interval of 126–165 ms (137 ± 11, *N* = 22).The notes of both single- and multiple-note calls consist of 3–8 pulses (5 ± 1, *N* = 74). Pulse duration is 1–2 ms (1.2 ± 0.4, *N* = 30), inter-pulse intervals are 1–2 ms (1.5 ± 0.4, *N* = 30). The high-pitched calls are emitted with a dominant frequency of 8,979–9,606 Hz (9,274 ± 195, *N* = 52) and have a bandwidth of 7328–11517 Hz (*N* = 33).

##### Distribution and natural history.

Our research team has sampled frogs at more than 150 permanent sampling sites distributed on both banks of the upper Madeira River and along the Purus-Madeira Interfluve. Yet, we have only observed *Dendropsophus bilobatus* in the lowland ombrophilous open forest on the east bank of the upper Madeira River. This area is close to the border between Brazil and Bolivia, and we expect that the new species also occurs in Bolivian lowland ombrophilous open forest, as do other anuran species that are known exclusively from the east bank of the upper Madeira River (e.g., *Hydrolaetare caparu* [Jansen, Gonzales-Álvarez & Köhler, 2007] and *Hamptophryne alios* [Wild, 1995]; Simões et al. 2011, Ferrão et al. 2014).

To date, specimens of *Dendropsophus bilobatus* have been observed only in the rainy season (early November to late March), which coincides with the species’ breeding season. Calling males were observed in flooded areas connected to rivers of moderate (Jaci-Parana River) to large size (Madeira River). Males typically call in a large chorus while perched on leaves and tiny trunks that range in height from just a few centimeters above the water surface to ~ 2 m high. Males start calling in the crepuscule (~ 18:00 hs) and call activity has been observed at least to approximately midnight. When call activity ends remains unknown. Amplexus has not been observed. Other sympatric frogs include *Rhaebo guttatus* (Schneider, 1799), *Boana cinerascens* (Spix, 1824), *B. lanciformis* (Cope, 1871), *Scinax* sp. 6 (sensu Ferrão et al. 2016) and an uncollected *Scinax* with an advertisement call that resembles that of *S. garbei* (Miranda-Ribeiro, 1926).

##### Etymology.

The specific name *bilobatus* is derived from the Latin noun *bilobate*. The name refers to the characteristic bilobate shape of the vocal sac of males of the new species.

## Discussion

The upper Madeira River is characterized by high biodiversity; several priority areas for conservation are identified in this region (Capobianco 2001). Yet, recent studies have revealed that knowledge of the species diversity of amphibians inhabiting forests in the upper Madeira River is still incomplete, and description of new species is ongoing (e.g., Simões et al. 2010; Brcko et al. 2013; Ferrão et al. 2016; Ferrão et al. 2018). The new taxon described herein is the first species of *Dendropsophus* described from the Brazilian portion of the upper Madeira River, and at least one other new *Dendropsophus* species is awaiting formal description (work in preparation). At the same time, many of these species are highly threatened by increasing levels of deforestation caused by both illegal expansion of pastureland and infrastructure development associated with human settlements (e.g., reconstruction of the BR-319 highway and construction of large hydroelectric powerplants: Fearnside and Graça 2006; Fearnside 2015). Forests adjacent to the type locality of *D. bilobatus* sp. nov. were illegally deforested in 2016 and the paratype locality is now surrounded by pastures.

Based on general morphological similarity with specimens in the type series, we tentatively refer specimens from Jirau-Direito and Morrinhos, two localities in the east bank of the upper Madeira River, to *D. bilobatus*. However, these specimens differ from the type material in some otherwise conserved characters (e.g., dorsal skin texture and color of iris), and there are no molecular data from them that might clarify their taxonomic relationship to the sequenced type specimens. Therefore, we have chosen not to include these specimens in the type series of *D. bilobatus* to avoid confounding specimens of the new species with what might turn out to be a second, but undescribed, cryptic species.

Intrageneric variation in vocal sac shape is not unusual in anurans, e.g., single subgular median in *Osteocephalus subtilis* and *O. oophagus*, single subgular expanded laterally in *O. vilarsi*, and paired lateral in *O. taurinus* (Jungfer and Schiesari 1995; Jungfer et al. 2013; Ferrão et al. 2019). As a result, vocal sac shape is a widely used character in anuran taxonomy and systematics. Aside from some members of the *Dendropsophus marmoratus* species group, *D. bilobatus* is the only species in the genus to possess a bilobate subgular vocal sac. All other congeners differ in the size and extent of a single subgular vocal sac, e.g., medium size in *D. bromeliaceus*, large size in *D. juliani*, well developed and extending laterally over the forearms in *D. ozzyi* (Moravec et al. 2006; Orrico et al. 2014; Ferreira et al. 2015). Given that the *D. marmoratus* and *D. microcephalus* species groups are not close relatives (Faivovich et al. 2005; Jansen et al. 2019; present study), the bilobate vocal sac evolved at least twice within *Dendropsophus*. However, this conclusion is tentative pending a fuller evaluation of the diversity and evolutionary history of vocal sac structures in *Dendropsophus* in a phylogenetic context.

Intrageneric differences in vocal sac shape have been associated with different breeding strategies in some neotropical anurans. For example, a small or indistinct single subgular vocal sac in phytotelmata-associated *Osteocephalus* is hypothesized to be a morphological adaptation for calling in small cavities relative to the large paired sacs of pond-breeding congeners (Jungfer and Hödl 2002; Moravec et al. 2009; Jungfer et al. 2013). We have not observed, however, any unusual feature of the breeding behavior or habitat of *Dendropsophus bilobatus* that might explain its remarkable bilobate vocal sac versus the single sac of most of its congeners.

## Supplementary Material

XML Treatment for Dendropsophus bilobatus

## Appendix I

Additional specimens used in comparisons.

*Dendropsophus* sp. A: BRAZIL: Amazonas: BR-319, RAPELD Module 11 (APL 19069-71, 20806); Rondônia: Porto Velho, RAPELD Teotônio (APL 22013, 22067).

*Dendropsophus* sp. B: BRAZIL: Rondônia: Porto Velho, RAPELD Bufalo (APL 19281, 20563), RAPELD Pedras (APL 19310, 19311, 20556, 20557).

*Dendropsophus aperomeus*: PERU: Amazonas: 8 km NNE of Balzapata (KU 181812 [holotype]); Huánuco: 30 km NE Tingo María, Cordillera Azul (AMNH 91917–18 [paratypes]); San Martín: Rioja: Venceremos, 89 km NW Rioja (KU 212085–98).

*Dendropsophus coffea*: BOLIVIA: La Paz: 55 km on road from Caranavi to Palos Blancos (NKA 6538 [holotype], ZFMK 80590 [paratype]); 5 km N Río Beni bridge, near Sapecho (CBF 5538, ZFMK 82182 [paratypes]).

*Dendropsophus delarivai*: BOLIVIA: Cochabamba: road from Paractito to Cochabamba via El Palmar (CBF 3332 [holotype], CBF 3331, CBF 3336–37, KU 224700, MNCN 23696–97, ZSM 1–3/1999, ZFMK 67139–42, ZFMK 70317 [paratypes]); La Paz: Colonia Eduardo Avaroa (NKA 6539, ZFMK 80587–88).

*Dendropsophus joannae*: BOLIVIA: Pando: Cobija (CBF 3323 [holotype], CBF 3324–26, KU 224701–03, ZFMK 67119–20, ZFMK 67121–24 [paratypes]); Nacebe (NMP6V 72169/1–2).

*Dendropsophus leali*: BOLIVIA: Beni: El Porvenir (CBF 2449–50, ZFMK 62826); Totaizal (CBF 2358–61); Bosque Chimanes (CBF 1859–60); Infierno Verde (CBF 1861–62); Puerto Almacén, Río Ibaré (ZFMK 60721–22); Rurrenabaque (CBF 1080); Cochabamba: 6.5 km N Chipiriri (KU 136281–94); Pando: Bolpebra (CBF 5806, NMP6V 72562). BRAZIL: Rondônia: Forte Príncipe da Beira (KU 92058–59 [paratypes]). PERU: Loreto: Puerto Almendras (NMP6V 71183); Madre de Dios: Cuzco Amazónico, 15 km E Puerto Maldonado (KU 205488–92, 205498–590, 207577–79).

*Dendropsophus mapinguari*: BRAZIL: Amazonas: kilometer 168 of the BR-319 federal highway, Purus-Madeira Interfluve (INPA-H 41071–72).

*Dendropsophus meridianus*: BRAZIL: Rio de Janeiro: 20 km N of Rio de Janeiro (ZFMK 39499–500).

*Dendropsophus microcephalus*: COSTA RICA: Guanacaste: Colorado (ZFMK 62142–48). VENEZUELA: Sucre: Parare (ZFMK 36085–94).

*Dendropsophus minimus*: BRAZIL: Amazonas: Taperinha (near Santarem) (NMW 19436 [holotype]).

*Dendropsophus minutus*: BOLIVIA: Chuquisaca: W of Vaca Guzmán (ZFMK 66045); Santa Cruz: Samaipata (ZFMK 60403–07); Laguna de Bermejo (ZFMK 60440); Pando: Barracón (NMP6V 72803/1–2); Bolpebra (NMP6V 72566); Cobija (NMP6V 72466, ZFMK 66790); Sena (NMP6V 72802/1–4).

*Dendropsophus miyatai*: PERU: Loreto: Anguilla (NMP6V 71259).

*Dendropsophus nanus*: BOLIVIA: Beni: Puerto Almacén (ZFMK 60458–62); 6.5 km NE of Riberalta (NMP6V 70693/1–3), 2 km SW of Riberalta (NMP6V 70694); Santa Cruz: Buenavista (ZFMK 80011–14); San Ramón (ZFMK 60391–92); La Florida (ZFMK 60374–81); Santa Cruz de la Sierra (ZFMK 67001). PARAGUAY: Chaco: 23 km S of Filadelfia (ZFMK 53262–66).

*Dendropsophus praestans*: COLOMBIA: Huila: Parque Arqueológico San Augustín (MCZ-A 100216 [paratype]).

*Dendropsophus riveroi*: BOLIVIA: Beni: El Trimefo (CBF 1960–90); road San Borja–Trinidad, Río Matos (CBF 2456–57); Totaizal (CBF 2691); Cobija (ZFMK 67145–48); Santa Cruz: Buenavista (ZFMK 80015–17). COLOMBIA: Amazonas: Leticia (CM 37433 [holotype]).

*Dendropsophus rhodopeplus*: BOLIVIA: Pando: Bioceanica (CBF 5813–14, NMP6V 72568); Bolpebra (NMP6V 72569). PERU: Loreto: Puerto Almendras (NMP6V 71179). BRAZIL: AMAZONAS: Porong, Rio Juruá (INPA-H 4006, 4010).

Dendropsophus cf. rubicundulus: BOLIVIA: La Paz: Puerto Moscoso, Laguna Piraña (CBF 5360–61).

Dendropsophus cf. schubarti: BOLIVIA: La Paz: La Paz: Puerto Moscoso, Laguna Piraña (CBF 5317).

*Dendropsophus tritaeniatus*: BOLIVIA: Santa Cruz: P.N. Noel Kempff Mercado (ZFMK 72688).

*Dendropsophus walfordi*: BRAZIL: Amazonas: Lago Catalão (INPA-H 25291–93); Rondônia: Forte Príncipe da Beira, Costa Marques (MZUSP 73652 [holotype], INPA-H 31321, 31324–25, 31331–32).

*Dendropsophus xapuriensis*: BOLIVIA: Pando: Bioceanica, (CBF 5684–89, NMP6V 72571/1–6).

## Appendix 2

**Table:** Species, voucher numbers, GenBank accession numbers, and localities of samples used for phylogenetic analyses.

Species	Voucher	GenBank	Locality	Reference
*D. berthalutzae*	CFBH5418	AY843607	Brazil: Rio de Janeiro, Duque de Caxias	Faivovich et al. (2005)
*D. bifurcus*	QCAZA23802	KY406446	Ecuador: Morona Santiago, 4 Km N de Macas	Caminer et al. (2017)
*D. bipunctatus*	MRT5946	AY843608	Brazil: Bahia, Jussari, Serra do Teimoso	Faivovich et al. (2005)
*D. brevifrons*	QCAZA48099	KT721783	Ecuador: Pompeya	Fouquet et al. (2015)
*D. cachimbo* A	MNKA9655	JF790046	Bolivia: Santa Cruz, Ñuflo de Chavez, San Sebastián	Jansen et al. (2011)
*D. cachimbo* A	MNKA9656	JF790047	Bolivia: Santa Cruz, Ñuflo de Chavez, San Sebastián	Jansen et al. (2011)
*D. carnifex*	QCAZA39333	KY406456	Ecuador: Ecuador: Imbabura, Santa Rosa	Caminer et al. (2017)
*D. coffea*	ZFMK82181	JF790050	Bolivia: La Paz, Sur Yungas, near Sapecho	Jansen et al. (2011)
*D. counani*	MNHN2015.107	KT721771	French Guiana: Montagne tortue grande	Fouquet et al. (2015)
*D. decipiens*	CFBHT07254	KU495203	Brazil: Sao Paulo, Cananeia	Lyra et al. (2017)
*D. elianeae*	ITH0653	AY843661	Brazil: Sao Paulo, Buri	Faivovich et al. (2005)
*D. gaucheri*	62BM	JF973303	French Guiana: Savane Corossony	Fouquet et al. (2011)
*D. gaucheri*	UTAA61327	JF973302	Suriname: Sipaliwini	Fouquet et al. (2011)
*D. juliani*	CBF5926	JF790052	Bolivia: Pando, Madre de Dios, Borracón	Jansen et al. (2011)
*D. juliani*	NMP6V72799/3	JF790051	Bolivia: Pando, Madre de Dios, Borracón	Jansen et al. (2011)
*D. juliani* A	MNKA9579	JF790053	Bolivia: Santa Cruz, Velasco, Caparu	Jansen et al. (2011)
*D. juliani* A	MNKA9919	JX187447	Bolivia: Santa Cruz, Velasco, Caparu	Schulze et al. (2015)
*D. leali*	MNKA9706	JF790057	Bolivia: Santa Cruz, Ñuflo de Chavez, San Sebastián	Jansen et al. (2011)
*D. leali*	MNKA10358	KF723024	Bolivia	Schulze et al. (2015)
*D. leucophyllatus*	MNHN2015.127	KY406356	French Guiana: Petit-saut	Caminer et al. (2017)
*D. luddeckei*	Chiquinquira03	JF422599	Colombia	Guarnizo et al. (2012)
*D. manonegra*	MHUAA7336	KF009943	Colombia: Caqueta, Florencia, vereda Sucre	Rivera-Correa and Orrico (2013)
*D. mathiassoni*	AJC1746	KP149479	Colombia: Meta, San Juan de Arama, Caserio Miraflores	Guarnizo et al. (2015)
*D. mathiassoni*	AJC3923	KP149474	Colombia: Meta, San Juan de Arama, Caserio Miraflores	Guarnizo et al. (2015)
*D. melanargyreus*	AS682	KF723036	Bolivia	Schulze et al. (2015)
*D. meridianus*		KM390784	Brazil	Chaves et al., unpublished
*D. microcephalus*	UTAA50632	AY843643	Honduras: Atlantida, Cordillera Nombre de Dios	Faivovich et al. (2005)
*D. microcephalus*	AJC4038	KP149404	Colombia: Santander, Reserva el arboretum	Guarnizo et al. (2015)
*D. microcephalus*	AJC3887	KP149423	Colombia: Santander, Sabana de Torres, Sabana de Torres	Guarnizo et al. (2015)
*D. minusculus*	48mc	EF376061	French Guiana	Salducci et al., unpublished
*D. minutus*	MNRJ77141	KJ833250	Brazil: Rio de Janeiro, Nova Friburgo	Gehara et al. (2014)
*D. miyatai*	JPC10772	AY843647	Ecuador: Sucumbios	Faivovich et al. (2005)
*D. nanus*	MNKA9474	JF790086	Bolivia: Santa Cruz, Sara, Buenavista	Jansen et al. (2011)
*D. nanus*	MACN37785	AY549346	Argentina: Entre Rios, Dto. Islas del Ibicuy	Faivovich et al. (2005)
*D. nanus*	SMF88421	JX187442	Bolivia: Santa Cruz, Ichilo, Buenavista	Schulze et al. (2015)
*D. novaisi*	ZUEC17858	KY053470	Brazil: Jequie, Bahia State	Teixeira et al. (2016b)
*D. reichlei*	EBT1	JF790109	Bolivia: Pando, Manuripi, Estación Biológica Tahuamanu	Jansen et al. (2011)
*D. reichlei*	EBT2	JF790108	Bolivia: Pando, Manuripi, Estación Biológica Tahuamanu	Jansen et al. (2011)
*D. rhodopeplus*	QCAZA44584	KY406466	Ecuador: Orellana, Huiririma	Caminer et al. (2017)
*D. rhodopeplus*	QCAZA44329	KY406465	Ecuador: Orellana, Chiroisla	Caminer et al. (2017)
*D. rozenmani*	MNKA9531	JF790112	Bolivia: Santa Cruz, Velasco, Caparu	Jansen et al. (2011)
*D. rozenmani*	MNKA9368	JF790115	Bolivia: Beni, Yucuma, Los Lagos	Jansen et al. (2011)
*D. sanborni*	MACN38638	AY843663	Argentina: Entre Rios, Dto. Islas del Ibicuy, Ruta 12 vieja	Faivovich et al. (2005)
*D. soaresi*	ZUEC16867	KY053471	Brazil: Barreiras, Bahia State	Teixeira et al. (2016b)
*D. walfordi*	MJH129	AY843683	Brazil	Faivovich et al. (2005)
*D. xapuriensis*	TG2812	KJ940034	Brazil: Acre, Tarauaca	Gehara et al. (2014)
*D. bilobatus* sp. nov.	**INPA-H 41301**	**MN977834**	Brazil: Rondonia, Porto Velho, Jaci Direito	This study
*D. bilobatus* sp. nov.	**INPA-H 41303**	**MN977835**	Brazil: Rondonia, Porto Velho, Jaci Novo	This study
*D. bilobatus* sp. nov.	**INPA-H 41305**	**MN977836**	Brazil: Rondonia, Porto Velho, Jaci Novo	This study
*D. bilobatus* sp. nov.	**INPA-H 41300**	**MN977837**	Brazil: Rondonia, Porto Velho, Jaci Novo	This study
*Dendropsophus* sp. A	**APL19069**	**MN977838**	Brazil: Amazonas, BR-319, RAPELD M11	This study
*Dendropsophus* sp. A	**APL19070**	**MN977839**	Brazil: Amazonas, BR-319, RAPELD M11	This study
*Dendropsophus* sp. A	**APL19071**	**MN977840**	Brazil: Amazonas, BR-319, RAPELD M11	This study
*Dendropsophus* sp. A	**APL20806**	**MN977841**	Brazil: Amazonas, BR-319, RAPELD M11	This study
*Dendropsophus* sp. A	**APL22013**	**MN977842**	Brazil: Rondônia, Porto Velho, RAPELD Teotônio	This study
*Dendropsophus* sp. A	**APL22067**	**MN977843**	Brazil: Rondônia, Porto Velho, RAPELD Teotônio	This study
*Dendropsophus* sp. B	**APL19281**	**MN977844**	Brazil: Rondônia, Porto Velho, RAPELD Bufalo	This study
*Dendropsophus* sp. B	**APL19310**	**MN977845**	Brazil: Rondônia, Porto Velho, RAPELD Pedras	This study
*Dendropsophus* sp. B	**APL19311**	**MN977846**	Brazil: Rondônia, Porto Velho, RAPELD Pedras	This study
*Dendropsophus* sp. B	**APL20556**	**MN977847**	Brazil: Rondônia, Porto Velho, RAPELD Pedras	This study
*Dendropsophus* sp. B	**APL20557**	**MN977848**	Brazil: Rondônia, Porto Velho, RAPELD Pedras	This study
*Dendropsophus* sp. B	**APL20563**	**MN977849**	Brazil: Rondônia, Porto Velho, RAPELD Bufalo	This study
*Xenohyla truncata*	CFBH7600	AY843775	Brazil: Rio de Janeiro, Restinga de Marica	Faivovich et al. (2005)
